# Gut inflammation associated with age and Alzheimer’s disease pathology: a human cohort study

**DOI:** 10.1038/s41598-023-45929-z

**Published:** 2023-11-14

**Authors:** Margo B. Heston, Kendra L. Hanslik, Katie R. Zarbock, Sandra J. Harding, Nancy J. Davenport-Sis, Robert L. Kerby, Nathaniel Chin, Yi Sun, Ana Hoeft, Yuetiva Deming, Nicholas M. Vogt, Tobey J. Betthauser, Sterling C. Johnson, Sanjay Asthana, Gwendlyn Kollmorgen, Ivonne Suridjan, Norbert Wild, Henrik Zetterberg, Kaj Blennow, Federico E. Rey, Barbara B. Bendlin, Tyler K. Ulland

**Affiliations:** 1 Wisconsin Alzheimer’s Disease Research Center, University of Wisconsin School of Medicine and Public Health, Madison, WI USA; 2 Department of Pathology and Laboratory Medicine, University of Wisconsin-Madison, Madison, WI USA; 3 Department of Bacteriology, University of Wisconsin-Madison, Madison, WI USA; 4 Wisconsin Alzheimer’s Institute, School of Medicine and Public Health, University of Wisconsin, Madison, WI USA; 5 Roche Diagnostics GmbH, Penzberg, Germany; 6 Roche Diagnostics International Ltd, Rotkreuz, Switzerland; 7 Department of Psychiatry and Neurochemistry, Institute of Neuroscience and Physiology, The Sahlgrenska Academy at the University of Gothenburg, Mölndal, Sweden; 8 Clinical Neurochemistry Laboratory, Sahlgrenska University Hospital, Mölndal, Sweden; 9 Department of Neurodegenerative Disease, UCL Institute of Neurology, London, UK; 10 UK Dementia Research Institute at UCL, London, UK; 11 Hong Kong Center for Neurodegenerative Diseases, Hong Kong, China

**Keywords:** Alzheimer's disease, Neurodegeneration

## Abstract

Age-related disease may be mediated by low levels of chronic inflammation (“inflammaging”). Recent work suggests that gut microbes can contribute to inflammation via degradation of the intestinal barrier. While aging and age-related diseases including Alzheimer’s disease (AD) are linked to altered microbiome composition and higher levels of gut microbial components in systemic circulation, the role of intestinal inflammation remains unclear. To investigate whether greater gut inflammation is associated with advanced age and AD pathology, we assessed fecal samples from older adults to measure calprotectin, an established marker of intestinal inflammation which is elevated in diseases of gut barrier integrity. Multiple regression with maximum likelihood estimation and Satorra–Bentler corrections were used to test relationships between fecal calprotectin and clinical diagnosis, participant age, cerebrospinal fluid biomarkers of AD pathology, amyloid burden measured using ^11^C-Pittsburgh compound B positron emission tomography (PiB PET) imaging, and performance on cognitive tests measuring executive function and verbal learning and recall. Calprotectin levels were elevated in advanced age and were higher in participants diagnosed with amyloid-confirmed AD dementia. Additionally, among individuals with AD dementia, higher calprotectin was associated with greater amyloid burden as measured with PiB PET. Exploratory analyses indicated that calprotectin levels were also associated with cerebrospinal fluid markers of AD, and with lower verbal memory function even among cognitively unimpaired participants. Taken together, these findings suggest that intestinal inflammation is linked with brain pathology even in the earliest disease stages. Moreover, intestinal inflammation may exacerbate the progression toward AD.

## Introduction

The gut microbiota is hypothesized to contribute to inflammaging, a condition characterized by chronic low-grade systemic inflammation without an acute driver such as infection, yet the physiological mechanisms by which this occurs remain poorly understood^1–5^. It is possible that age-related shifts in the composition of the microbiome produce an inflammatory environment within the intestine, which degrades the epithelial barrier. This may enable luminal bacterial components such as lipopolysaccharides and peptidoglycan fragments to enter systemic circulation and trigger a peripheral inflammatory response^2,6–8^. Evidence supports the barrier degradation component of this hypothesis, suggesting that gut permeability increases with aging and age-related diseases. For instance, levels of high-mobility group box 1 protein, which induces intestinal inflammation, are correlated with advanced age as well as markers of chronic inflammation, frailty, and impaired nutrient absorption^9–11^. Further, elevated permeability appears more frequently among older adults with inflammatory conditions including type 2 diabetes and irritable bowel syndrome (IBS)^2,12^. However, few intestinal permeability studies have been completed in healthy aging adults, and these studies have yielded inconclusive evidence, so it remains unclear whether elevated inflammation and permeability can occur prior to the onset of age-related disease.

Among age-related neurodegenerative diseases, mounting evidence suggests that gut dysbiosis and peripheral inflammation are associated with Alzheimer’s disease (AD) and may contribute to AD pathogenesis^13–17^. AD, which is defined by cerebral aggregation of amyloid beta (Aβ) and phosphorylated tau (pTau) proteins, is accompanied by microglial and astrocytic activation and associated neuroinflammation^18–21^. Factors including gut dysbiosis and gut bacteria derived metabolites have been linked to inflammation, preclinical cognitive decline, and AD diagnosis, but their mechanistic relationships with pathology remain ill-defined^16,17,22–26^. A recent study found that IBS was associated with higher risk of developing AD dementia^27^. As IBS may induce intestinal permeability via microbial dysbiosis, this suggests a possible role for intestinal inflammation and permeability in AD^12^. These prior observations and gaps in current knowledge motivated the current evaluation of intestinal inflammation in aging and as a precursor to AD pathology.

To index gut inflammation, we collected fecal samples from middle-aged and older adults along the biologic and clinical AD continuum and measured fecal calprotectin, a marker of intestinal inflammation. Calprotectin is a heterodimer of S100 calcium-binding proteins expressed primarily in myeloid cells and released from the cytosol of activated neutrophils^28,29^. This molecular complex is suitable as a biomarker due to its abundance in neutrophils, constituting approximately 60% of their cytosolic protein, and its stability at room temperature for up to 7 days^30–32^. We tested associations of fecal calprotectin with years of age, cognitive function, and markers of AD pathology indexed by positron emission tomography (PET) neuroimaging and cerebrospinal fluid (CSF) biomarkers. We obtained measurements from participants across the AD continuum, and classified three disease stages using clinical diagnosis and amyloid positivity: cognitively unimpaired and Aβ-negative (CU Aβ−), cognitively unimpaired and Aβ-positive (CU Aβ+), and AD dementia with confirmed Aβ positivity (AD Aβ+). Linear regression was used to test the extent to which intestinal inflammation was associated with age, cerebral amyloid retention, and cognitive decline. Here we found that calprotectin levels were higher in participants diagnosed with amyloid-confirmed AD dementia and, consistent with inflammaging, calprotectin was increased with age, even among cognitively unimpaired participants. Individuals with AD Aβ+ had higher levels of calprotectin correlated with greater amyloid burden, and across the full cohort there were associations with fluid biomarkers of AD pathology. Finally, among cognitively unimpaired participants we found that calprotectin levels were associated with subtle cognitive changes that are linked to future onset of AD.

## Materials and methods

### Recruitment and clinical methods

Participants were recruited from the Wisconsin Alzheimer’s Disease Research Center (ADRC) Clinical Core and the Wisconsin Registry for Alzheimer’s Prevention (WRAP)^33^, two prospective cohort studies that enroll adults starting in middle age. Upon enrollment, participants underwent comprehensive cognitive evaluation, interviews of medical and family history, and *APOE* genotyping. Participants also underwent longitudinal cognitive assessments and laboratory testing, and a subset underwent lumbar puncture and/or ^11^C-Pittsburgh compound B (PiB) PET neuroimaging to quantify AD pathological burden. Participants in this cohort were additionally co-enrolled in the Microbiome in Alzheimer’s Disease Risk Study (MARS), which collects and analyzes fecal samples to determine the role of the gut microbiota in AD development^17^. Participants with a banked fecal sample, cognitive assessment, and confirmation of amyloid status within two years of a fecal sample were included in the study.

Clinical diagnosis of cognitively unimpaired, mild cognitive impairment due to AD, or AD dementia was determined via multidisciplinary consensus panel in accordance with the 2011 National Institute on Aging-Alzheimer’s Association (NIA-AA) workgroup diagnostic criteria^34,35^. Amyloid status was determined using CSF collected at lumbar puncture or PET neuroimaging procedure that was closest in time to the date of fecal sample collection. Amyloid positivity was indexed by: global PiB distribution volume ratio (DVR) greater than 1.19, ^18^F-AV45 PET positivity assessed using visual inspection by an expert rater (SCJ), or CSF Aβ_42_/Aβ_40_ ratio less than 0.046^36,37^. Rating of amyloid status was performed with respect to the biomarker variables alone, blind to all other experimental variables.

All participants received laboratory testing including height and weight measurements, which were used to calculate body mass index (BMI), and routine blood draws. As previously published, *APOE* ε2/ε3/ε4 genotyping was performed at baseline using allele-specific polymerase chain reaction-based assays, and for the present study a pseudo-continuous *APOE* risk score was calculated using estimates derived from neuropathologically confirmed cases in the Alzheimer’s Disease Neuroimaging Initiative^33,38^. At the time that fecal samples were obtained, participants completed questionnaires detailing current diet and medications, history of gastrointestinal and cardiovascular disease, and lifestyle factors that alter gut microbiome composition, including early childhood microbial exposures and current exposure to house pets or other animals^17^.

### Biomarker measurement using CSF and PiB PET

CSF was acquired using the Wisconsin ADRC CSF Biomarker service procedures, typically collected in the morning to control for diurnal variability^36^. Biomarkers were measured using a robust prototype assay as part of the Roche NeuroToolKit research platform (Aβ_42_/Aβ_40_, pTau_181_/Aβ_42_, total tau [tTau]), glial activation (chitinase-3-like protein 1 [YKL-40], glial fibrillary acidic protein [GFAP], soluble triggering receptor expressed on myeloid cells 2 [sTREM2], S100 calcium binding protein B [S100B]), axonal degeneration (neurofilament light protein [NfL]), synaptic degeneration (neurogranin, α-synuclein), and inflammation (IL-6). In this study, CSF IL-6 was employed as a marker related to systemic inflammation. In vivo animal model studies show that peripheral inflammation contributes to neuroinflammation in AD, and in healthy volunteers, experimental stimulation with *E. coli*-derived lipopolysaccharide (LPS) resulted in increased IL-6 levels in both blood and CSF^39,40^. This suggests that CSF IL-6 levels may reflect peripheral inflammation. CSF biomarkers were quantified using Elecsys® assays for Aβ_42_, pTau_181_, and tTau. These, along with S100B and IL-6, were performed on a cobas e® 601 analyzer, and assays for Aβ_40_, YKL-40, GFAP, sTREM2, NfL, neurogranin, and α-synuclein were performed on a cobas e® 411 analyzer^33,36^.

PiB PET scans were acquired, processed, and quantified using published methods^37^. Briefly, participants underwent PET (Siemens EXACT HR+) imaging procedures at the University of Wisconsin-Madison Waisman Center. PiB DVR was estimated using reference Logan graphical analysis, and a global cortical DVR average was calculated using DVR from eight bilateral regions that exhibit Aβ burden early in preclinical AD. Regions were determined using the Automated Anatomical Labelling Atlas 3 and included the anterior cingulate cortex, angular gyrus, middle temporal gyrus, posterior cingulate cortex, precuneus, supramarginal gyrus, superior temporal gyrus, and ventromedial prefrontal cortex.

### Neuropsychological assessment

Participants underwent the Rey Auditory Verbal Learning Test (RAVLT) to assess verbal learning and memory, and the Trail Making Test (TMT) part B to assess executive function^41,42^. These measures are standards used to assess cognitive decline starting in preclinical AD; the TMT is part of the National Alzheimer’s Coordinating Center Uniform Data Set, and the RAVLT is widely used in large studies of cognitive aging^43–47^. Raw scores were used as outcome measures in regression analyses and models controlled for age, sex, educational attainment, and amyloid status. The RAVLT Trials 1–5 score denotes the total words learned over 5 repetitions of a 15-word list (range = 0–75), and the RAVLT Trial 7 score denotes the total words recalled from this list after a delay (range = 0–15). The TMT Trial B score indicates the participant’s time in seconds to complete drawing a continuous trail that alternates between ascending numbered and lettered icons printed on the page.

### Fecal collection, processing, and calprotectin measurement

Fecal samples were collected using published methods^17^. Briefly, participants completed sample collection at home, recording the time of collection and returning the samples to the University of Wisconsin-Madison in insulated containers with frozen gel packs via overnight delivery. Upon arrival, samples were promptly processed and frozen by an individual blinded to the subjects’ demographics. Table 1 and Tables S2–S5 describe times between sample collection and freezing as the sample collection-storage interval. Samples were weighed and scored using the Bristol Stool Scale (BSS), a measure of stool consistency. Representative samples of approximately 2–4 g of fecal material were obtained using two sterile straws; both straws were placed in a 15 ml conical tube and stored at −80 °C until processing. A subset of samples remained in their collection receptacles without representative sampling.

**Table 1:** Participant demographics.

Characteristic	CU Aβ−, N = 79*	CU Aβ+, N = 33*	AD Aβ+, N = 13*	*P*-values			
				Global	CU Aβ− vs. CU Aβ+	CU Aβ− vs. AD Aβ+	CU Aβ+ vs. AD Aβ+
Age at fecal sample, *years*	65.92 (5.97)	68.92 (6.34)	73.90 (5.09)	< 0.001^†^	0.06^§^	< .001^§^	0.03^§^
Sex				0.9^††^			
Female	50 (63%)	22 (67%)	9 (69%)		–	–	–
Male	29 (37%)	11 (33%)	4 (31%)		–	–	–
*APOE* genotype				< 0.001^††^			
ε2–ε3	11 (13.9%)	1 (3.0%)	0 (0%)		0.7^††^	> 0.9^††^	> 0.9^††^
ε2–ε4	1 (1.3%)	1 (3.0%)	0 (0%)		0.7^††^	> 0.9^††^	> 0.9^††^
ε3–ε3	43 (54.4%)	14 (42%)	2 (15.4%)		–	–	–
ε3–ε4	19 (24.1%)	15 (45.5%)	7 (53.8%)		0.2^††^	0.06^††^	0.06^††^
ε4–ε4	1 (1.3%)	2 (6.1%)	4 (30.8%)		0.4^††^	0.004^††^	0.1^††^
Unknown	4	0	0		–	–	–
Education, *years*	16.41 (2.71)	16.18 (2.26)	14.69 (2.02)	0.03^†^	0.8^§^	0.03^§^	0.1^§^
BMI, *kg/m*^*2*^	28 (4.9)	28 (5.2)	26 (3.6)	0.4^†^	–	–	–
Fecal sample Bristol Score	3.9 (1.2)	3.8 (1.2)	3.9 (1.4)	> 0.9^†^	–	–	–
Sample collection-storage interval, *days*	1.1 (0.31)	1.1 (0.46)	1.4 (1.1)	0.6^†^	–	–	–
Unknown	1	0	0				

*Mean (SD); n (%).

^†^One-way analysis of means (not assuming equal variances).

^††^Fisher's exact test.

^§^Games–Howell test.

Bristol Stool Scale (BSS) ranges from 1 (hard lumps) to 7 (liquid). *P*-values from pairwise Fisher’s and Games–Howell tests are Benjamini–Hochberg corrected, per characteristic. For pairwise Fisher’s tests on *APOE* data, ε3–ε3 homozygotes were the reference group.

The concentration of calprotectin was measured using an enzyme-linked immunosorbent assay (ELISA; Eagle Biosciences, catalog no. CAL35-K01). For each subject, calprotectin levels were measured using aliquots from two distinct locations within the fecal sample. Samples were read at 450 nm with a reference filter at 620 nm, and a cubic equation was used to form a standard curve and calculate signal intensities. Based on clinical tests of the assay that established control and elevated ranges, calprotectin levels were considered elevated when greater than 43.2 μg/g (or 1.6 log(μg/g) after linearizing the ELISA absorbance distribution; see “Statistical methods” for details)^48^. All tests were conducted in duplicate in a blinded manner.

### Statistical methods

To obtain the average calprotectin level for each participant, mean values were calculated using the two fecal sample aliquots. Mean calprotectin levels subsequently underwent a positive translation of 11.3 µg/g and a log_10_ transformation to linearize the absorbance distribution of the ELISA. Multiple linear regression was used to test the extent to which calprotectin level varied with (1) age and diagnosis/amyloid positivity, (2) global PiB DVR, (3) CSF biomarker levels, and (4) tests of cognitive function among CU participants. Calprotectin-by-disease stage interaction predictors were included for global PiB DVR models, upon visual inspection of the bivariate relationships between calprotectin and biomarker/cognitive outcomes. One model was used per outcome, for a total of 2 age and disease stage models, 1 PiB PET model, 11 CSF biomarker models, and 3 cognitive testing models. When modeling calprotectin as an outcome, covariates included participant sex and BMI, as these are known to relate to the gut microbiome, which modulates intestinal and peripheral inflammatory processes^49^. For models involving PET, CSF or cognitive outcomes, covariates included factors known to correlate with AD pathology, including age, sex, amyloid-confirmed consensus diagnosis, BMI, and *APOE* risk score. Full models were tested initially; in cases where effects were found nonsignificant in the full models, a model building approach was employed to clarify the sources of variance. The magnitude and significance of the effects of interest are reported across the model building steps in Tables S6, S7, and S9. Regressions were performed in the R package *lavaan*, using maximum likelihood estimation and Satorra–Bentler corrections to adjust for heterogeneity of model residuals^50,51^. Benjamini–Hochberg adjustments were used to control for inflated Type I error rate; adjusted *P*-values are denoted as *Q*-values^52^. For effects that were found to be significant (Q < 0.05) among the full cohort, models were re-evaluated to determine whether results remained significant among the CU participant subset.

To identify factors that were associated with elevated calprotectin among CU Aβ− participants, post hoc analyses were performed to compare microbiome-altering lifestyle factors, medications, and early life exposure events. Participants with calprotectin levels greater than 1.6 log(μg/g) were assigned to the high-calprotectin group, and the remaining participants were assigned to the low-calprotectin group. Wilcoxon rank sum and Fisher exact tests were used to test factors across the two calprotectin groups.

### Ethics approval and informed consent to participate

Prior to admission into the WRAP, Wisconsin ADRC, and MARS studies, participants provided written informed consent in accordance with the Declaration of Helsinki. All work was completed under approved protocols of the University of Wisconsin-Madison Institutional Review Board (WRAP and Wisconsin ADRC umbrella protocol ID: 2013-0178; MARS protocol ID: 2015-1121).

## Results

### Participant demographics

125 participants (CU Aβ−, n = 79; CU Aβ+, n = 33; AD Aβ+, n = 13) provided samples which were assessed for levels of fecal calprotectin. Across biologic and clinical AD stages, participant groups did not significantly differ in sex composition or mean BMI, and fecal samples did not differ in mean BSS score or in time interval between sample collection and storage. Educational attainment was generally high, however participants with AD Aβ+ had comparably lower years of education (P = 0.03). CU Aβ− participants had completed a mean ± standard deviation (SD) of 16.41 ± 2.71 years of education and CU Aβ+ participants had completed 16.18 ± 2.26 years (equivalent to a bachelor’s degree), while AD Aβ+ participants had completed 14.69 ± 2.02 years (associate degree). Participants with AD Aβ+ were older on average (mean ± SD: 73.9 ± 5.09 years) compared with CU Aβ− (65.92 ± 5.97 years) and CU Aβ+ (68.92 ± 6.34 years) participants. AD Aβ+ participants were enriched for homozygous *APOE* ε4 carriage (30.8%) compared to CU Aβ− participants (1.3%). Table 1 describes the demographics for the full cohort, and Table S1 compares the medication usage between disease stage; medications were identified using the Anatomical Therapeutic Chemical classification system^53^. Tables S2–S5 feature the demographics for each subset in analyses using global PiB DVR, CSF biomarkers, and neuropsychological assessment as outcomes. Each participant subset was compositionally representative of the full cohort. Neuropsychological assessment scores were comparable to those from the parent WRAP and ADRC cohorts, and they were slightly better than previously reported values in other aging studies^33,44,54^.

### Calprotectin is higher in older participants and those with AD Aβ+

**Figure 1:**
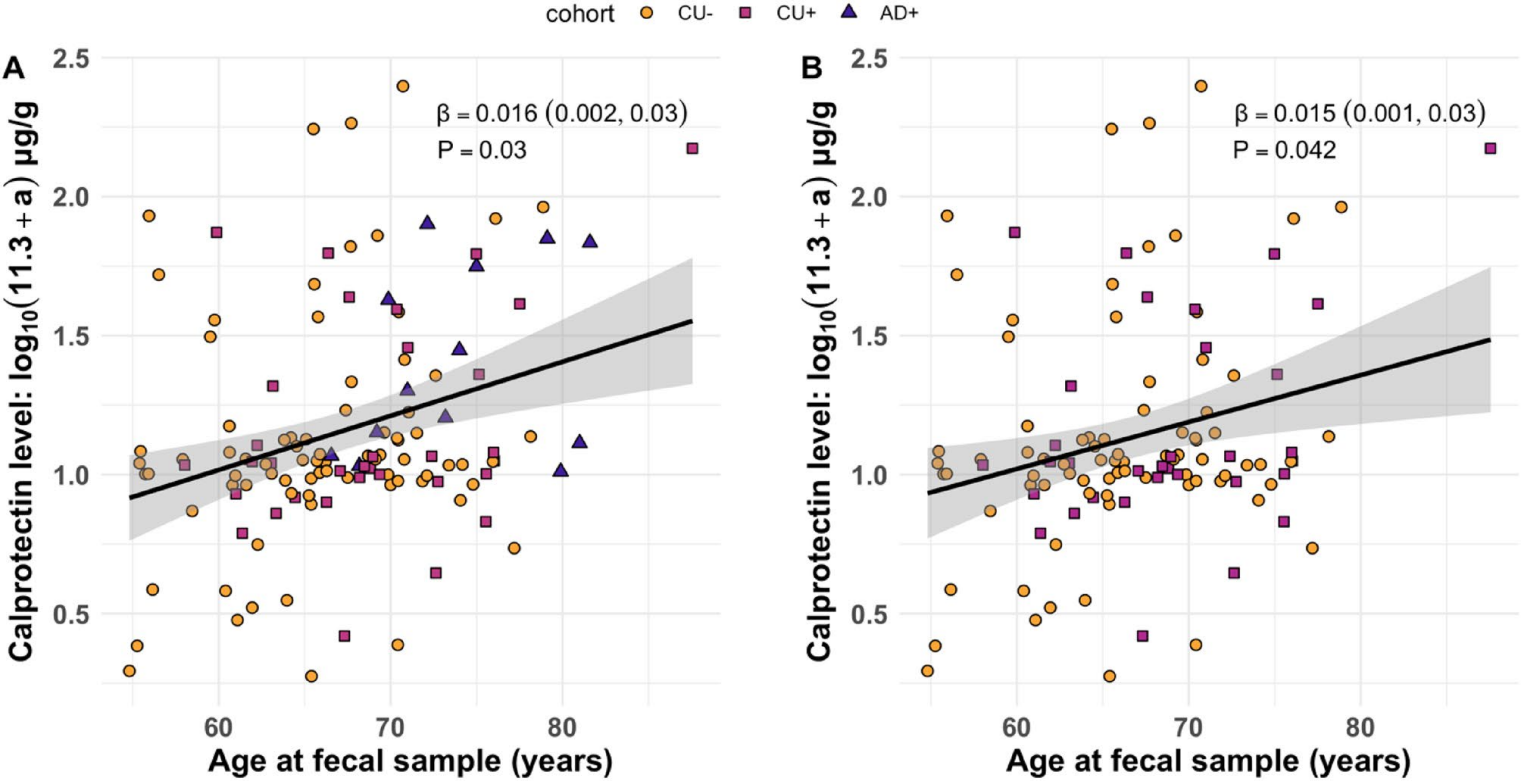
Calprotectin association with age. (**A**) Relationship between participant age and fecal calprotectin levels across all disease statuses. (**B**) Relationship between participant age and fecal calprotectin levels before cognitive decline. *β* coefficients (multiple regression) were controlled for disease stage, sex, and BMI.

**Figure 2:**
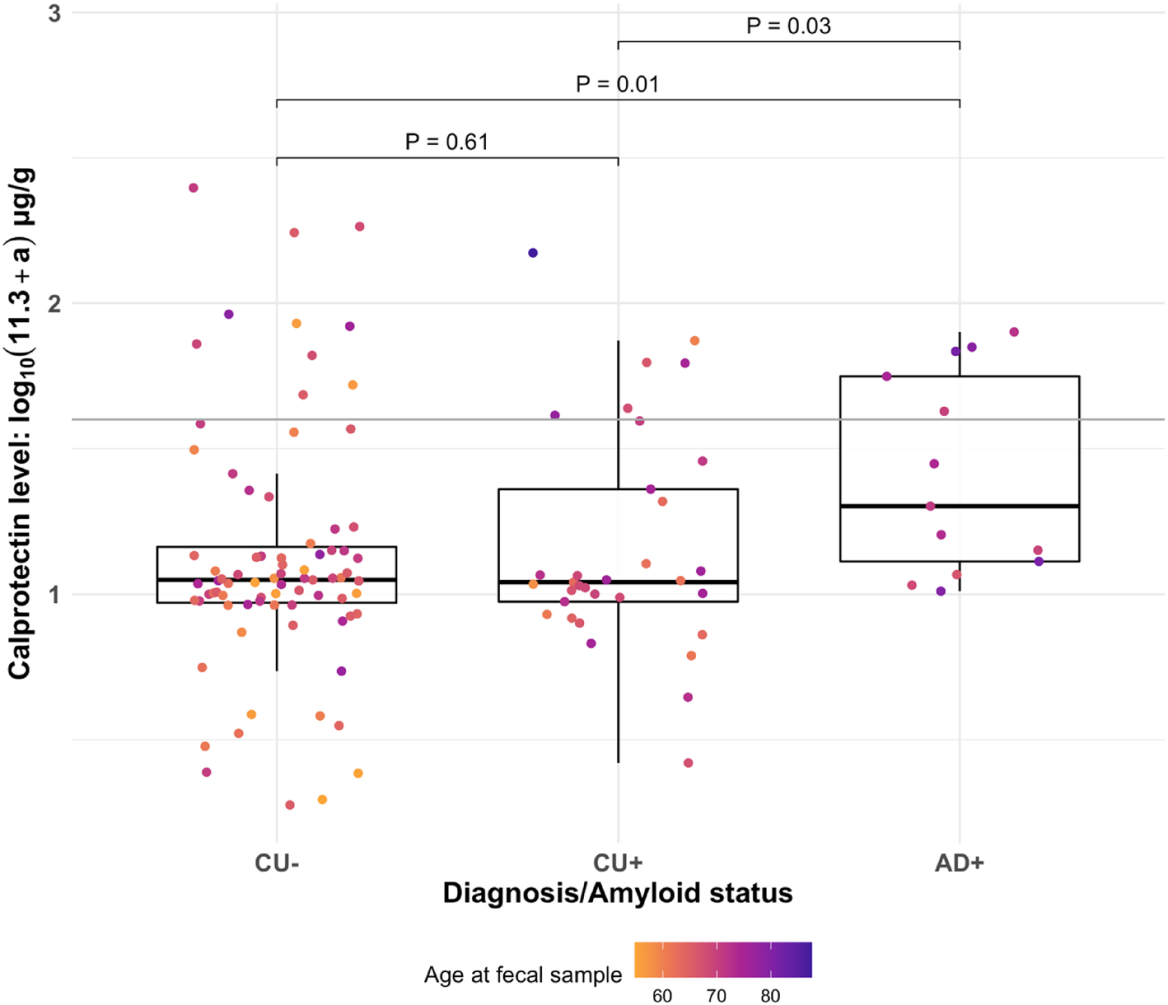
Calprotectin association with disease status. Fecal calprotectin is considered elevated at 43.2 μg/g or 1.6 log(μg/g), denoted by the gray horizontal line and defined by the ELISA standard curve. *P*-values (multiple regression) are uncontrolled for covariates.

To test the hypothesis that intestinal inflammation increases with age and AD progression, fecal calprotectin was regressed on participant age at fecal sample and on disease stage (CU Aβ−, CU Aβ+, AD Aβ+). Across the full cohort, advanced age was significantly associated with greater calprotectin (*β* [95% CI] = 0.016 [0.002, 0.03], P = 0.027), controlling for effects of disease stage, sex, and BMI (Fig. 1A). This effect was sustained upon restricting analysis to participants with a CU consensus diagnosis (0.015 [6.0e−4, 0.03], P = 0.042) (Fig. 1B). Regression effects for the full cohort are tabulated in Table S6, and effects for CU participants alone are tabulated in Table S7. In addition, before adjusting for age, mean calprotectin was significantly higher among AD Aβ+ participants compared with CU Aβ− participants (0.28 [0.08, 0.49], P = 0.010) and with CU Aβ+ participants (0.24 [0.02, 0.47], P = 0.030) (Fig. 2).

While age and AD Aβ+ status were associated with greater calprotectin, visual inspection of calprotectin plots revealed that several CU Aβ− participants (n = 10) exhibited elevated calprotectin relative to the high-calprotectin threshold of the ELISA, which was not significantly associated with advanced age (P = 0.40, Table S8). To identify factors that distinguished the high-calprotectin CU Aβ− participants from their low-calprotectin counterparts, a post hoc analysis was conducted on self-reported comorbidities, medications, dietary patterns, and other microbiome-altering lifestyle factors^55–57^. Of the factors examined, there were no differences in prevalence of reported IBD, IBS, and probiotic usage. Only proton pump inhibitor (PPI) usage was identified as being more frequent among high-calprotectin CU Aβ− participants (P = 0.013, Table S8), with 4 in 10 of these participants reporting use of PPI.

### Calprotectin-by-AD Aβ+ interaction on global PiB DVR

**Figure 3:**
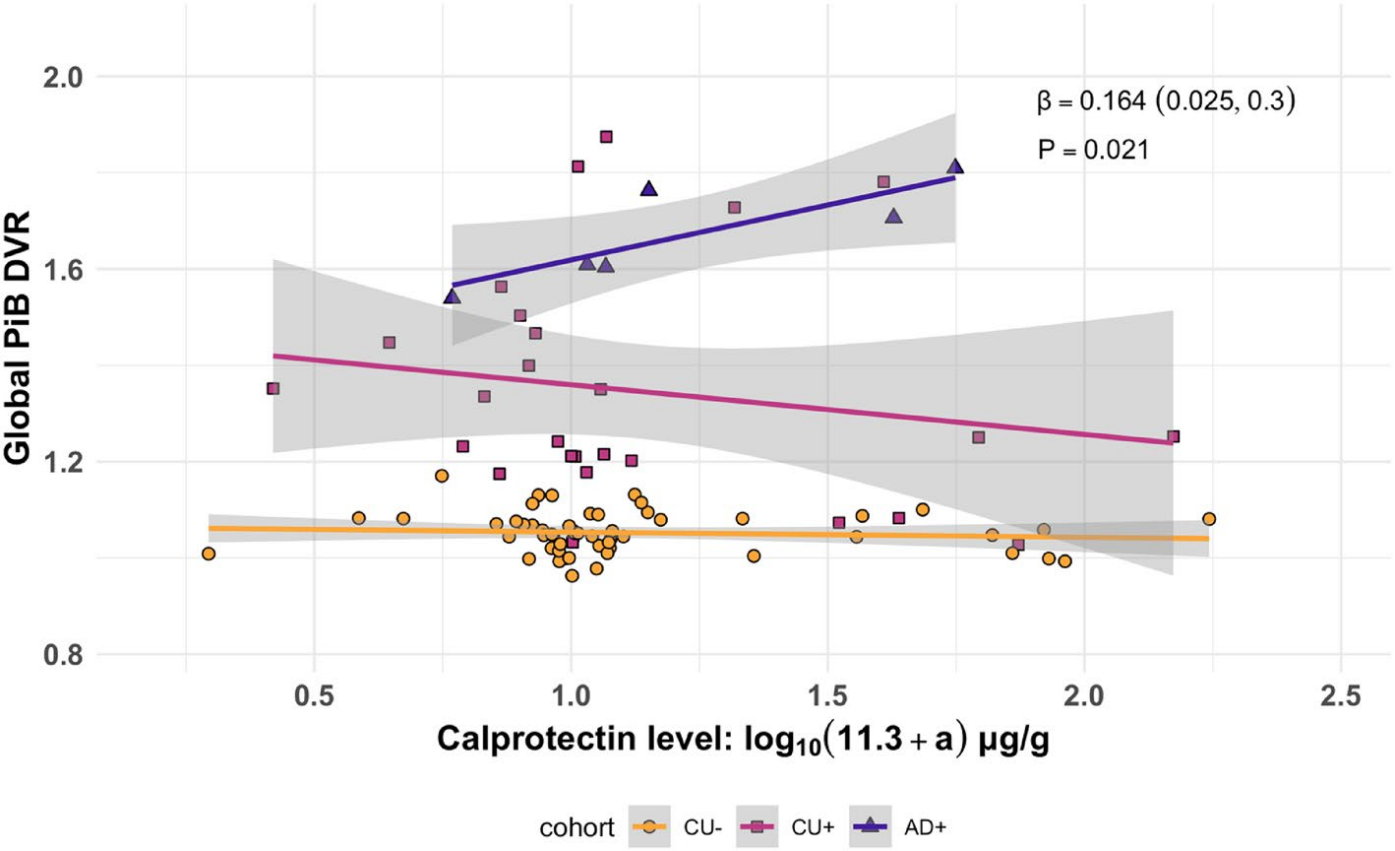
Calprotectin-by-AD dementia interaction effect on global PiB DVR. *β* coefficient and *P*-value were estimated using the multiple regression equation $DVR=calprotectin+disease \, status+\left(calprotectin\times disease \, status\right)+age+APOE+sex+BMI$.

The main effects of gut inflammation and the interaction between gut inflammation and disease status on brain Aβ burden were tested using global PiB DVR. Eighty-seven individuals with fecal calprotectin, disease status, and PiB PET neuroimaging collected within 2 years of a fecal sample were included. Regression analysis showed that global PiB DVR was positively associated with calprotectin within the AD Aβ+ participant subset; this effect remained significant after adjustment for age, sex, *APOE* risk score and BMI (P = 0.021, Fig. 3). Among CU Aβ+ participants, calprotectin did not show a significant relationship with global PiB DVR (P = 0.62, Table S9). To test the extent to which disease stage interactions were confounded by age, global PiB DVR models were re-evaluated using a calprotectin-by-age interaction term. The calprotectin-by-age effect was found nonsignificant (P = 0.17) even before controlling for covariates. This suggested that calprotectin had a pathology-specific relationship with amyloid, independent from the effect of age (Table S9).

### CSF biomarkers and cognitive function variance with calprotectin level

**Figure 4:**
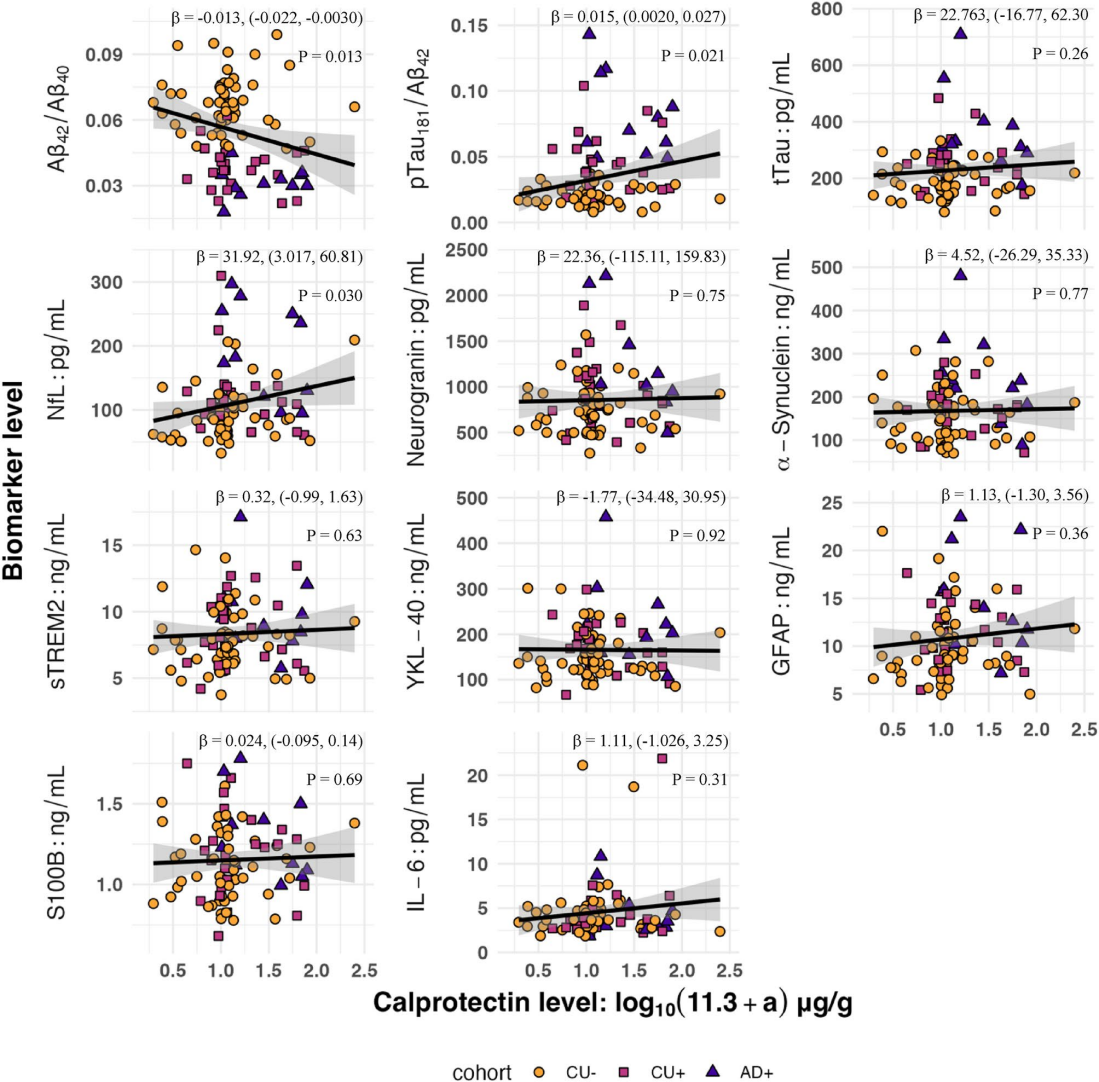
CSF biomarker links to fecal calprotectin. *β* coefficients and *P*-values (multiple regression) were estimated without controlling for covariates.

We conducted exploratory analyses of calprotectin and CSF biomarkers of AD, glial activation, and neurodegeneration^58^. Ninety-one participants with CSF biomarkers within 2 years of a fecal sample were included, and 10 CSF biomarkers related to AD pathology, glial activation, axonal degeneration, synaptic degeneration, and inflammation were separately regressed on fecal calprotectin. Prior to adding covariates, higher calprotectin was significantly associated with lower Aβ_42_/Aβ_40_, higher pTau_181_/Aβ_42_, and higher NfL concentration (P = 0.013, P = 0.021, and P = 0.030, respectively) (Fig. 4). However, these effects were not significant after correction for multiple comparisons or adjustment for participant age and disease stage (Table S10).

To test the hypothesis that intestinal inflammation is associated with lower cognitive function before diagnosis of AD Aβ+, the RAVLT Trials 1–5 (word recall immediately post-learning), RAVLT Trial 7 (delayed word recall), and TMT part B (executive function) assessments were separately regressed on fecal calprotectin. One hundred and three CU participants with cognitive assessments within 2 years of a fecal sample were included. Higher calprotectin was associated with lower delayed recall (fewer words remembered in RAVLT trial 7), controlling for educational attainment and amyloid status. Effects of calprotectin on cognitive testing performance were not significant after controlling for age and multiple comparisons adjustment (Fig. S1).

## Discussion

Inflammaging is a chronic, low-grade inflammation associated with age which may be driven by alterations in the composition of the gut microbiome and by a reduced selectivity of the intestinal barrier^59^. Prior evidence suggests that age-related gut microbiome disruptions may elicit intestinal and systemic inflammation that contributes to diseases, potentially including neurodegenerative diseases such as AD, however the relationships between age, gut permeability, and disease markers have not been well established^16,17,25,59^. To evaluate the extent to which intestinal inflammation correlates with age and markers of AD, we measured fecal calprotectin levels in CU Aβ−, CU Aβ+, and AD Aβ+ individuals, and found that intestinal inflammation was associated with age, AD Aβ+ status, cortical amyloid among individuals with AD Aβ+, and CSF biomarkers of AD and neurodegeneration.

As a biomarker, calprotectin was selected due to its cytosolic abundance in neutrophils, its stability at room temperature, and its involvement in inflammatory disease. Calprotectin is a marker specific to inflammation, as several in vitro and clinical studies indicate that calprotectin plays a role in monocyte recruitment^60–62^. In diagnostic practice, fecal calprotectin has shown utility as a marker of intestinal inflammation. Notably, fecal calprotectin distinguishes between inflammatory bowel disease (IBD), which is marked by increased gut permeability, from functional gastrointestinal disorders^63–65^. Together, these studies support fecal calprotectin’s utility to reflect conditions marked by intestinal permeability.

While there are few existing human studies on calprotectin, emerging evidence suggests that fecal calprotectin levels may vary across the life course and in age-related disease^66–70^. For instance, in a population study of children aged 0 to 18 years old, median fecal calprotectin level among healthy infants under 1 year old was nearly twice that of children between 1 and 18 years old^71–74^. One small study showed a two- to tenfold difference in fecal calprotectin among 45 healthy participants between 30 and 80 years old, however the vast majority of aging studies have examined calprotectin among cohorts with clinical disease diagnoses^64,66,69,70,75^. While our study was enriched for individuals with biologic and clinical AD, we determined that older participants had higher levels of fecal calprotectin while controlling for disease stage, and that the results remained significant after performing sensitivity analyses that included only CU participants. This suggests that advancing age increases gut inflammation independently of symptomatic AD.

Only one prior study evaluated fecal calprotectin among 22 participants with AD Aβ+, finding that calprotectin was elevated in this cohort^75^. Similarly, we found that participants in our study with AD Aβ+ had higher calprotectin. Additionally, we leveraged in vivo biomarkers including CSF assays and amyloid PET to better determine the relationship between intestinal inflammation and AD pathology. We found that higher calprotectin was associated with greater cortical amyloid burden among AD Aβ+ participants, an effect that was independent of age. Calprotectin also nominally correlated with CSF Aβ_42_/Aβ_40_, pTau_181_/Aβ_42_, and NfL. Both CSF Aβ_42_/Aβ_40_ and pTau_181_/Aβ_42_ are markers specific to AD, with the latter biomarker incorporating both amyloid and pTau pathology, while NfL is a marker of axonal degeneration. Taken together, these findings suggest that intestinal inflammation is also associated with neurodegeneration.

While it may be the case that intestinal inflammation exacerbates the progression of AD, given that the analyses conducted here were cross-sectional, we cannot rule out the possibility that development of AD pathology may exacerbate intestinal inflammation. However, it is interesting to note that even among CU participants, we observed a relationship between intestinal inflammation and memory function. Previously, it was observed that cognitive dysfunction was associated with intestinal inflammation in other conditions such as Crohn’s disease and IBS^76,77^. These conditions were excluded from the current study; accordingly, calprotectin levels in our study were generally lower than compared with the levels observed among individuals with these conditions. Taken together with prior studies on IBS and IBD, our results showing relationships between calprotectin and memory function suggest that interventions that mitigate intestinal inflammation could have a beneficial impact on cognitive function in older adults.

In addition to associations of calprotectin with age and AD pathology, the present study also suggested that elevated intestinal inflammation may be present among otherwise healthy adults who are negative for amyloid pathology (CU Aβ−). A greater proportion of high-calprotectin participants reported using PPI medications. PPIs are associated with lower microbiome α-diversity, higher abundances of genera including *Enterococcus*, and lower abundances of genera including *Bifidobacterium*^57^. *Enterococcus faecalis* has been shown to generate neurofibrillary epitopes in rat cortical neurons, while *Bifidobacterium* species are differentially abundant in AD compared with controls^17,78,79^. *Bifidobacterium* species produce various molecules that may ameliorate risk factors for AD, including angiotensin I-converting enzyme inhibiting peptides which could be used to reduce blood pressure, and short-chain fatty acids that contribute to intestinal barrier maintenance^80–82^. Apart from PPI usage, there were no disease-related trends which explained the high calprotectin observed in fecal samples of CU Aβ− participants. Identifying elevated calprotectin among otherwise healthy participants may provide an opportunity to determine associations with later disease development and may reveal points of intervention to restore intestinal barrier function. Future longitudinal studies are needed to determine whether high intestinal inflammation predicts greater pathology and accelerated cognitive decline at subsequent time points.

While the present study has elucidated age and AD relationships with calprotectin, some limitations should be noted. The research cohorts included in this study are lacking in diversity (participants were predominantly White and highly educated) and may not reflect the broader population. Continuous sampling of the enrolled individuals will enable longitudinal studies of gut inflammation effects on AD. Additionally, active efforts to recruit community members will enable future studies of the intestinal barrier in a more diverse aging population. This study is also limited in that it did not include gut microbiome data, blood-based biomarkers of inflammation, or blood measures of microbial translocation including LPS. These data would enable evaluation of the connected hypotheses that gut microbiome alterations may increase intestinal permeability, contributing to systemic inflammation or microbial interactions with the blood–brain barrier, and thereby exacerbating AD pathology. Surveying the microbiome’s structure and function, bacterial translocation into the periphery, and mediating effects on the observed calprotectin-amyloid relationship will be essential for understanding the microbiome’s role in intestinal permeability and potential downstream effects on AD pathology. While a blood biomarker of inflammation was unavailable, CSF IL-6 provided an indirect measure for this study. CSF and plasma levels of IL-6 are correlated in conditions involving neuronal injury (e.g., subarachnoid hemorrhage) and chronic systemic inflammation (e.g., fibromyalgia), and a prior study using an experimental challenge with bacterial LPS showed tandem increases in CSF and peripheral blood IL-6 in humans, suggesting that CSF IL-6 may at least partially reflect peripheral inflammation^39,83,84^. In the present study, CSF IL-6 showed a positive nonsignificant trend with calprotectin levels, warranting future re-evaluation with IL-6 measured in blood.

While measuring fecal calprotectin enabled us to capture inflammation in the large intestine, the effects of the small intestine on aging and AD pathology remain unknown. Emerging evidence from human studies suggests that the small intestine exhibits microbial alterations in aging and AD, and increased permeability with age. For instance, a study evaluating the duodenal microbiome of individuals from 18 to 80 years old noted a reduction of microbial diversity and an increase of *Escherichia*, *Lactobacillus*, and *Enterococcus* genera with age^85^. Among a small cohort, individuals with AD were found to have higher rates of small intestinal bacterial overgrowth, which is interrelated with IBS and hypothesized to induce intestinal permeability via microbial dysbiosis^12,70,86,87^. Separately, reductions in the ionic gradient of the small intestine have been noted with age^12^. In future studies we plan to examine small intestinal permeability and gut microbiome composition as it relates to age and preclinical AD pathology.

## Conclusions

Our findings provide evidence that aging may co-occur with intestinal inflammation, that intestinal inflammation is observed in AD Aβ+ and may exacerbate amyloid accumulation, and that associations with cognitive function may appear even prior to development of symptomatic AD Aβ+. Together, these results suggest that intestinal inflammation and possible accompanying intestinal permeability could be modifiable targets in aging and AD.

### Supplementary Information

Supplementary Information.

## Data Availability

Data generated and analyzed during the current study via the Wisconsin ADRC and WRAP protocols are available on reasonable request via an online REDCap request tool, which can be accessed at https://www.adrc.wisc.edu/apply-resources. Questions regarding the availability of data can be addressed to ADRCrequest@medicine.wisc.edu. To further discuss data management please contact Dr. Yue Ma (yma@medicine.wisc.edu).

## Abbreviations

Aβ: Amyloid-beta
AD: Alzheimer’s disease
ADRC: Alzheimer’s Disease Research Center
*APOE*: Apolipoprotein E
BMI: Body mass index
CSF: Cerebrospinal fluid
CU: Cognitively unimpaired
DVR: Distribution volume ratio
GFAP: Glial fibrillary acidic protein
IBD: Inflammatory bowel disease
IBS: Irritable bowel syndrome
MARS: Microbiome in Alzheimer’s Study
NIA-AA: National Institute on Aging-Alzheimer’s Association
NfL: Neurofilament light protein
PET: Positron emission tomography
PiB: ^11^C-Pittsburgh compound B
PPI: Proton pump inhibitor
pTau: Phosphorylated tau
RAVLT: Rey auditory verbal learning test
S100B: S100 calcium binding protein B
sTREM2: Soluble triggering receptor expressed on myeloid cells 2
TMT: Trail making test
tTau: Total tau
WRAP: Wisconsin Registry for Alzheimer’s Prevention
YKL-40: Chitinase-3-like protein 1
