# Supplementary material for: Gut inflammation associated with age and Alzheimer’s disease pathology: a human cohort study
Source: Sci Rep. 2023 Nov 14;13:18924. doi: 10.1038/s41598-023-45929-z (PMC10646035; doi:10.1038/s41598-023-45929-z)
Supplement: Supplementary file 1 — Supplementary Information. [file 41598_2023_45929_MOESM1_ESM.docx]

# **Supplementary Materials**

## **
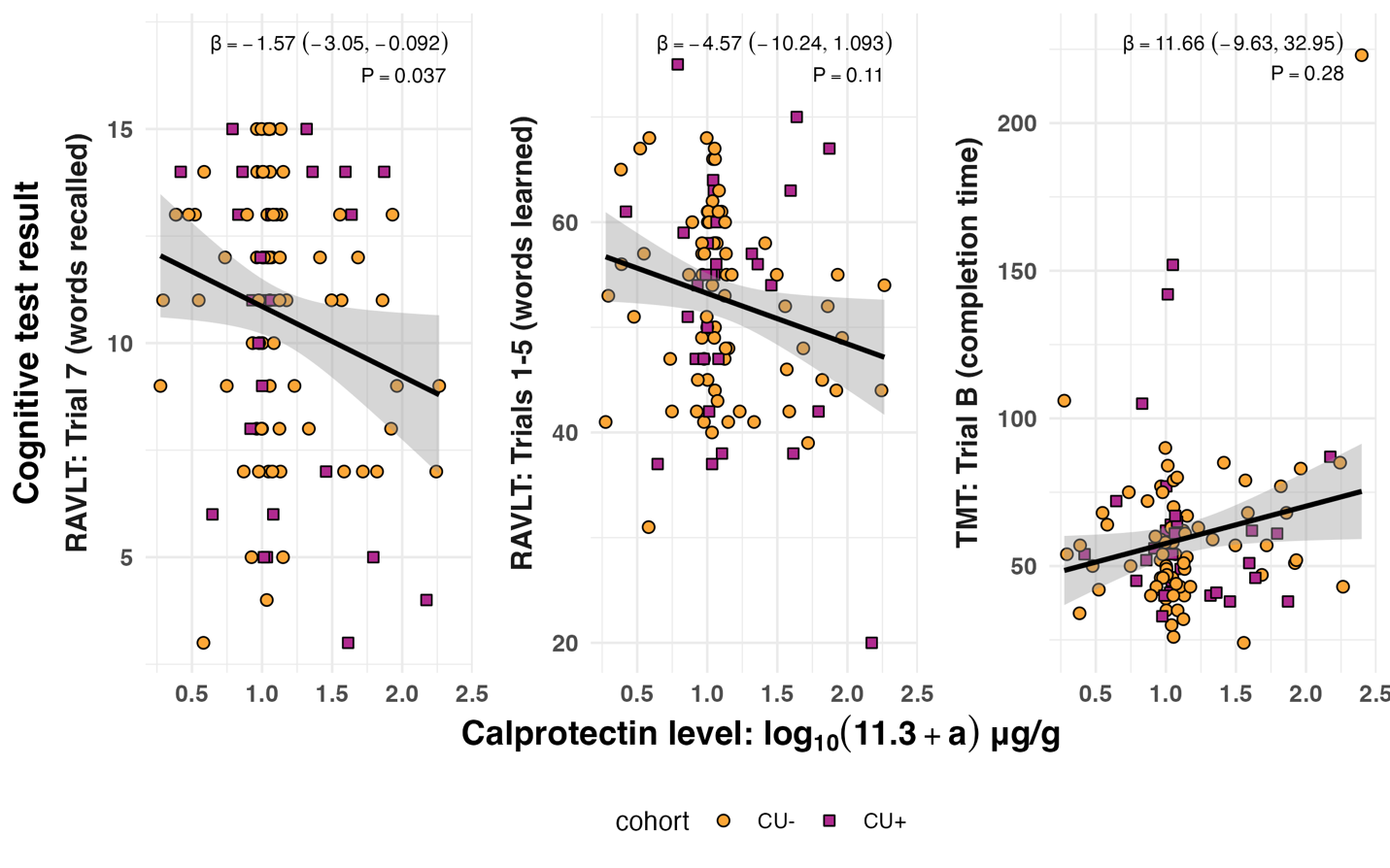
Fig. S1.** **Calprotectin effects on cognitive performance in cognitively unimpaired participants.** *CU Aβ-*, cognitively unimpaired, Aβ-negative; *CU Aβ+*, cognitively unimpaired, Aβ-positive; *RAVLT*, Rey Auditory Verbal Learning Test; *TMT*, Trail Making Test. β coefficients (multiple regression, *p*-values false discovery rate uncorrected) are reported with adjustment for education and disease status; no effects survived adjustment for age or sex covariates. An outlier was noted among the TMT Trial B; removal of outlier did not alter the direction or significance of the reported effect.

**Table S1. Participant medication usage.**

|  |  |  |  |  | | ***p*-values** | | | | | | |
| --- | --- | --- | --- | --- | --- | --- | --- | --- | --- | --- | --- | --- |
| **Characteristic** | **ATC code(s)** | **CU Aβ-,  N = 74^*^** | **CU Aβ+,  N = 31^*^** | **AD Aβ+,  N = 13^*^** | | **Global** | | **CU Aβ- vs. CU+** | | **CU Aβ- vs. AD Aβ+,** | | **CU Aβ+ vs. AD Aβ+,** |
| ACE inhibitors | C09AA | 8 (11%) | 4 (13%) | 0 (0%) | | 0.5^†^ | | – | | – | | – |
| Alendronic acid | M05BA04 | 0 (0%) | 1 (3.2%) | 2 (15%) | | 0.010^†^ | | 0.30^†^ | | 0.06^†^ | | 0.30^†^ |
| Alpha-blockers | G04CA | 3 (4.1%) | 1 (3.2%) | 1 (7.7%) | | 0.6^†^ | | – | | – | | – |
| Amitriptyline | N06AA09 | 2 (2.7%) | 1 (3.2%) | 0 (0%) | | >0.9^†^ | | – | | – | | – |
| Amlodipine | C08CA01 | 1 (1.4%) | 2 (6.5%) | 1 (7.7%) | | 0.2^†^ | | – | | – | | – |
| Amoxicillin | J01CA04 | 1 (1.4%) | 0 (0%) | 1 (7.7%) | | 0.3^†^ | | – | | – | | – |
| Anticholinesterases | N06DA | 0 (0%) | 0 (0%) | 11 (85%) | | <0.001^†^ | | >0.9^†^ | | <0.001^†^ | | <0.001^†^ |
| Anticoagulants | B01A | 34 (46%) | 13 (42%) | 7 (54%) | | 0.8^††^ | | – | | – | | – |
| Ascorbic acid | A11GA01 | 6 (8.1%) | 0 (0%) | 0 (0%) | | 0.3^†^ | | – | | – | | – |
| B vitamins | A11D | 6 (8.1%) | 1 (3.2%) | 0 (0%) | | 0.6^†^ | | – | | – | | – |
| Benazepril and amlodipine | C09BB13 | 0 (0%) | 1 (3.2%) | 0 (0%) | | 0.4^†^ | | – | | – | | – |
| Benzodiazepines | N05BA N03AE | 6 (8.1%) | 1 (3.2%) | 2 (15%) | | 0.3^†^ | | – | | – | | – |
| Benzonatate | R05DB01 | 1 (1.4%) | 0 (0%) | 0 (0%) | | >0.9^†^ | | – | | – | | – |
| Beta agonists | R03AC | 4 (5.4%) | 1 (3.2%) | 0 (0%) | | >0.9^†^ | | – | | – | | – |
| Beta-blockers | C07A | 13 (18%) | 3 (9.7%) | 1 (7.7%) | | 0.5^†^ | | – | | – | | – |
| Bulk-forming laxatives | A06AC | 1 (1.4%) | 0 (0%) | 0 (0%) | | >0.9^†^ | | – | | – | | – |
| Biotin | A11HA05 | 3 (4.1%) | 1 (3.2%) | 0 (0%) | | >0.9^†^ | | – | | – | | – |
| Botulinum toxin | M03AX01 | 0 (0%) | 1 (3.2%) | 0 (0%) | | 0.4^†^ | | – | | – | | – |
| Brimonidine | S01EA05 | 1 (1.4%) | 0 (0%) | 0 (0%) | | >0.9^†^ | | – | | – | | – |
| Bupropion | N06AX12 | 3 (4.1%) | 0 (0%) | 0 (0%) | | 0.7^†^ | | – | | – | | – |
| Calcium | A12AA | 24 (32%) | 12 (39%) | 5 (38%) | | 0.8^†^ | | – | | – | | – |
| Carbamazepine | N03AF01 | 1 (1.4%) | 0 (0%) | 0 (0%) | | >0.9^†^ | | – | | – | | – |
| Carbamide | D02AE51 | 0 (0%) | 0 (0%) | 1 (7.7%) | | 0.11^†^ | | – | | – | | – |
| Celecoxib | M01AH01 | 1 (1.4%) | 0 (0%) | 0 (0%) | | >0.9^†^ | | – | | – | | – |
| Cetirizine | R06AE07 | 3 (4.1%) | 0 (0%) | 0 (0%) | | 0.7^†^ | | – | | – | | – |
| Chondroitin sulfate | M01AX25 | 2 (2.7%) | 2 (6.5%) | 0 (0%) | | 0.7^†^ | | – | | – | | – |
| Chlortalidone | C03BA04 | 1 (1.4%) | 0 (0%) | 0 (0%) | | >0.9^†^ | | – | | – | | – |
| Ciclosporin | S01XA18 | 0 (0%) | 1 (3.2%) | 0 (0%) | | 0.4^†^ | | – | | – | | – |
| Colecalciferol | A11CC05 | 28 (38%) | 7 (23%) | 5 (38%) | | 0.3^†^ | | – | | – | | – |
| Collagenase | D03BA02 | 0 (0%) | 0 (0%) | 1 (7.7%) | | 0.11^†^ | | – | | – | | – |
| Corticosteroids | D07AA D07AB | 2 (2.7%) | 0 (0%) | 0 (0%) | | >0.9^†^ | | – | | – | | – |
| Cyanocobalamin | B03BA01 | 2 (2.7%) | 1 (3.2%) | 2 (15%) | | 0.13^†^ | | – | | – | | – |
| Cyclobenzaprine | M03BX08 | 1 (1.4%) | 0 (0%) | 0 (0%) | | >0.9^†^ | | – | | – | | – |
| Diphenhydramine | R06AA02 | 4 (5.4%) | 1 (3.2%) | 0 (0%) | | >0.9^†^ | | – | | – | | – |
| Docusate sodium | A06AA02 | 2 (2.7%) | 0 (0%) | 0 (0%) | | >0.9^†^ | | – | | – | | – |
| Dopamine agonists | N04BC | 2 (2.7%) | 0 (0%) | 0 (0%) | | >0.9^†^ | | – | | – | | – |
| Dorzolamide | S01EC03 | 1 (1.4%) | 0 (0%) | 0 (0%) | | >0.9^†^ | | – | | – | | – |
| Enzyme preparations | A09AA | 3 (4.1%) | 0 (0%) | 0 (0%) | | 0.7^†^ | | – | | – | | – |
| Estrogens | G03CA | 5 (6.8%) | 2 (6.5%) | 0 (0%) | | >0.9^†^ | | – | | – | | – |
| Ezetimibe | C10AX09 | 0 (0%) | 2 (6.5%) | 0 (0%) | | 0.14^†^ | | – | | – | | – |
| Fenofibrate | C10AB05 | 1 (1.4%) | 0 (0%) | 0 (0%) | | >0.9^†^ | | – | | – | | – |
| Ferrous sulfate | B03AA07 | 2 (2.7%) | 2 (6.5%) | 0 (0%) | | 0.7^†^ | | – | | – | | – |
| Finasteride | G04CB01 | 1 (1.4%) | 1 (3.2%) | 0 (0%) | | 0.6^†^ | | – | | – | | – |
| Flecainide | C01BC04 | 0 (0%) | 0 (0%) | 1 (7.7%) | | 0.11^†^ | | – | | – | | – |
| Fluticasone | R03BA05 | 2 (2.7%) | 1 (3.2%) | 0 (0%) | | >0.9^†^ | | – | | – | | – |
| Fluticasone propionate | R01AD08 | 1 (1.4%) | 1 (3.2%) | 0 (0%) | | 0.6^†^ | | – | | – | | – |
| Folic acid | B03BB01 | 2 (2.7%) | 0 (0%) | 0 (0%) | | >0.9^†^ | | – | | – | | – |
| Gabapentin | N02BF01 | 3 (4.1%) | 0 (0%) | 1 (7.7%) | | 0.3^†^ | | – | | – | | – |
| Ginkgo folium | N06DX02 | 3 (4.1%) | 0 (0%) | 0 (0%) | | 0.7^†^ | | – | | – | | – |
| Glimepiride | A10BB12 | 1 (1.4%) | 0 (0%) | 0 (0%) | | >0.9^†^ | | – | | – | | – |
| Glucosamine | M01AX05 | 10 (14%) | 2 (6.5%) | 0 (0%) | | 0.4^†^ | | – | | – | | – |
| H_2_ antagonists | A02BA | 2 (2.7%) | 2 (6.5%) | 0 (0%) | | 0.7^†^ | | – | | – | | – |
| Hydrochlorothiazide | C03AA03 | 3 (4.1%) | 4 (13%) | 2 (15%) | | 0.14^†^ | | – | | – | | – |
| Indometacin | M01AB01 | 0 (0%) | 0 (0%) | 1 (7.7%) | | 0.11^†^ | | – | | – | | – |
| Insulin | A10AB | 2 (2.7%) | 0 (0%) | 0 (0%) | | >0.9^†^ | | – | | – | | – |
| Ipratropium bromide | R03BB01 | 1 (1.4%) | 0 (0%) | 0 (0%) | | >0.9^†^ | | – | | – | | – |
| Levocarnitine | A16AA01 | 1 (1.4%) | 0 (0%) | 0 (0%) | | >0.9^†^ | | – | | – | | – |
| Levonorgestrel | G03AC03 | 1 (1.4%) | 0 (0%) | 0 (0%) | | >0.9^†^ | | – | | – | | – |
| Loperamide | A07DA03 | 1 (1.4%) | 0 (0%) | 0 (0%) | | >0.9^†^ | | – | | – | | – |
| Loratadine | R06AX13 | 1 (1.4%) | 1 (3.2%) | 1 (7.7%) | | 0.2^†^ | | – | | – | | – |
| Losartan | C09CA01 | 6 (8.1%) | 1 (3.2%) | 2 (15%) | | 0.3^†^ | | – | | – | | – |
| Losartan and diuretics | C09DA01 | 1 (1.4%) | 1 (3.2%) | 0 (0%) | | 0.6^†^ | | – | | – | | – |
| Lysine | B05XB03 | 1 (1.4%) | 0 (0%) | 0 (0%) | | >0.9^†^ | | – | | – | | – |
| Magnesium supplements | A12CC | 3 (4.1%) | 1 (3.2%) | 0 (0%) | | >0.9^†^ | | – | | – | | – |
| Melatonin | N05CH01 | 5 (6.8%) | 0 (0%) | 0 (0%) | | 0.4^†^ | | – | | – | | – |
| Meloxicam | M01AC06 | 2 (2.7%) | 1 (3.2%) | 0 (0%) | | >0.9^†^ | | – | | – | | – |
| Memantine | N06DX01 | 0 (0%) | 0 (0%) | 7 (54%) | | <0.001^†^ | | >0.9^†^ | | <0.001^†^ | | <0.001^†^ |
| Metformin | A10BA02 | 4 (5.4%) | 0 (0%) | 0 (0%) | | 0.6^†^ | | – | | – | | – |
| Metronidazole | D06BX01 | 0 (0%) | 1 (3.2%) | 0 (0%) | | 0.4^†^ | | – | | – | | – |
| Mirtazapine | N06AX11 | 0 (0%) | 0 (0%) | 1 (7.7%) | | 0.11^†^ | | – | | – | | – |
| Montelukast | R03DC03 | 1 (1.4%) | 1 (3.2%) | 0 (0%) | | 0.6^†^ | | – | | – | | – |
| Multivitamins | A11BA A11AA | 29 (39%) | 12 (39%) | 7 (54%) | | 0.6^††^ | | – | | – | | – |
| Nabumetone | M01AX01 | 2 (2.7%) | 0 (0%) | 0 (0%) | | >0.9^†^ | | – | | – | | – |
| Nonsteroidal anti-inflammatory drugs | M01AE | 14 (19%) | 3 (9.7%) | 2 (15%) | | 0.6^†^ | | – | | – | | – |
| Omega-3 triglycerides | C10AX06 | 20 (27%) | 9 (29%) | 2 (15%) | | 0.7^†^ | | – | | – | | – |
| Osmotic laxatives | A06AD | 3 (4.1%) | 1 (3.2%) | 0 (0%) | | >0.9^†^ | | – | | – | | – |
| Paracetamol | N02BE01 | 3 (4.1%) | 2 (6.5%) | 1 (7.7%) | | 0.5^†^ | | – | | – | | – |
| Potassium gluconate | A12BA05 | 1 (1.4%) | 1 (3.2%) | 0 (0%) | | 0.6^†^ | | – | | – | | – |
| Progesterone | G03DA04 | 2 (2.7%) | 0 (0%) | 0 (0%) | | >0.9^†^ | | – | | – | | – |
| Prostaglandin analogues | S01EE | 4 (5.4%) | 0 (0%) | 0 (0%) | | 0.6^†^ | | – | | – | | – |
| Proton pump inhibitors | A02BC | 6 (8.1%) | 6 (19%) | 4 (31%) | | 0.041^†^ | | 0.26^†^ | | 0.12^†^ | | 0.44^†^ |
| Pseudoephedrine | R01BA02 | 1 (1.4%) | 0 (0%) | 0 (0%) | | >0.9^†^ | | – | | – | | – |
| Psychostimulants | N06BA | 1 (1.4%) | 0 (0%) | 1 (7.7%) | | 0.3^†^ | | – | | – | | – |
| Quinine | M09AA72 | 1 (1.4%) | 0 (0%) | 0 (0%) | | >0.9^†^ | | – | | – | | – |
| Rizatriptan | N02CC04 | 1 (1.4%) | 0 (0%) | 0 (0%) | | >0.9^†^ | | – | | – | | – |
| Selective serotonin reuptake inhibitors | N06AB | 9 (12%) | 4 (13%) | 7 (54%) | | 0.004^†^ | | >0.9^†^ | | 0.005^†^ | | 0.01^†^ |
| Selenium | A12CE | 1 (1.4%) | 0 (0%) | 0 (0%) | | >0.9^†^ | | – | | – | | – |
| Serotonin-norepinephrine reuptake inhibitors | N06AX | 4 (5.4%) | 1 (3.2%) | 0 (0%) | | >0.9^†^ | | – | | – | | – |
| Sildenafil | G04BE03 | 2 (2.7%) | 2 (6.5%) | 0 (0%) | | 0.7^†^ | | – | | – | | – |
| Silicones | A03AX13 | 1 (1.4%) | 0 (0%) | 0 (0%) | | >0.9^†^ | | – | | – | | – |
| Sodium fluoride | A01AA01 | 0 (0%) | 0 (0%) | 1 (7.7%) | | 0.11^†^ | | – | | – | | – |
| Spironolactone | C03DA01 | 1 (1.4%) | 0 (0%) | 0 (0%) | | >0.9^†^ | | – | | – | | – |
| Statins | C10AA | 28 (38%) | 11 (35%) | 10 (77%) | | 0.022^††^ | | >0.9^†^ | | 0.03^†^ | | 0.030^†^ |
| Temazepam | N05CD07 | 1 (1.4%) | 0 (0%) | 0 (0%) | | >0.9^†^ | | – | | – | | – |
| Testosterone | G03BA03 | 1 (1.4%) | 0 (0%) | 0 (0%) | | >0.9^†^ | | – | | – | | – |
| Thioctic acid | A16AX01 | 1 (1.4%) | 0 (0%) | 0 (0%) | | >0.9^†^ | | – | | – | | – |
| Thyroid hormones | H03AA | 18 (24%) | 4 (13%) | 3 (23%) | | 0.4^†^ | | – | | – | | – |
| Topiramate | N03AX11 | 1 (1.4%) | 0 (0%) | 0 (0%) | | >0.9^†^ | | – | | – | | – |
| Trazodone | N06AX05 | 0 (0%) | 2 (6.5%) | 1 (7.7%) | | 0.071^†^ | | – | | – | | – |
| Tretinoin | D10AD01 | 0 (0%) | 1 (3.2%) | 0 (0%) | | 0.4^†^ | | – | | – | | – |
| Triamterene | C03DB02 | 1 (1.4%) | 0 (0%) | 0 (0%) | | >0.9^†^ | | – | | – | | – |
| Ubidecarenone | C01EB09 | 6 (8.1%) | 1 (3.2%) | 1 (7.7%) | | 0.7^†^ | | – | | – | | – |
| Urinary incontinence drugs | G04BD | 3 (4.1%) | 0 (0%) | 0 (0%) | | 0.7^†^ | | – | | – | | – |
| Valaciclovir | J05AB11 | 0 (0%) | 1 (3.2%) | 0 (0%) | | 0.4^†^ | | – | | – | | – |
| Vitamin D and analogues | A11CC | 3 (4.1%) | 3 (9.7%) | 0 (0%) | | 0.4^†^ | | – | | – | | – |
| Vitamin E | A11HA03 | 3 (4.1%) | 0 (0%) | 2 (15%) | | 0.10^†^ | | – | | – | | – |
| Vitamin K | B02BA | 1 (1.4%) | 0 (0%) | 0 (0%) | | >0.9^†^ | | – | | – | | – |
| Zinc gluconate | A12CB02 | 1 (1.4%) | 0 (0%) | 0 (0%) | | >0.9^†^ | | – | | – | | – |
| Zolpidem | N05CF02 | 1 (1.4%) | 1 (3.2%) | 0 (0%) | | 0.6^†^ | | – | | – | | – |
| ^*^n (%) | | | | |  | |  | |  | |  |  |
| ^†^Fisher's exact test  ^††^Pearson's Chi-squared test | | | | |  | |  | |  | |  |  |

*AD Aβ+*, Alzheimer’s disease dementia, Aβ-positive; *ATC,* Anatomical Therapeutic Chemical classification system; *CU Aβ-*, cognitively unimpaired, Aβ-negative; *CU Aβ+*, cognitively unimpaired, Aβ-positive. Posthoc pairwise Fisher’s exact tests were Bonferroni-Hochberg corrected for Type I inflation error due to multiple comparisons testing.

**Table S2. Participant characteristics across all outcome measures.**

| **Characteristic** | **Amyloid confirmation, N = 125**^*^ | **Global PiB DVR, N = 87**^*^ | **CSF biomarkers, N = 90**^*^ | **Cognitive testing, N = 103**^*^ | ***p*-value** | |
| --- | --- | --- | --- | --- | --- | --- |
| Diagnosis/amyloid confirmation | |  |  |  | 0.039^††^ | |
| CU Aβ- | 79 (63%) | 55 (63%) | 54 (60%) | 73 (71%) |  | |
| CU Aβ+ | 33 (26%) | 26 (30%) | 25 (28%) | 30 (29%) |  | |
| AD Aβ+ | 13 (10%) | 6 (6.9%) | 11 (12%) | 0 (0%) |  | |
| Age at fecal sample, *years* | 67.54 (6.46) | 67.47 (6.14) | 67.16 (6.61) | 66.61 (6.38) | 0.7^†^ | |
| Sex |  |  |  |  | >0.9^†^ | |
| Female | 81 (65%) | 57 (66%) | 56 (62%) | 65 (63%) |  | |
| Male | 44 (35%) | 30 (34%) | 34 (38%) | 38 (37%) |  | |
| *APOE* genotype |  |  |  |  |  | |
| E2-E3 | 12 (9.9%) | 8 (9.2%) | 9 (10%) | 10 (10%) |  | |
| E2-E4 | 2 (1.7%) | 0 (0%) | 2 (2.3%) | 2 (2.0%) |  | |
| E3-E3 | 59 (49%) | 42 (48%) | 38 (44%) | 55 (56%) |  | |
| E3-E4 | 41 (34%) | 32 (37%) | 32 (37%) | 31 (31%) |  | |
| E4-E4 | 7 (5.8%) | 5 (5.7%) | 5 (5.8%) | 1 (1.0%) |  | |
| Unknown | 4 | 0 | 4 | 4 |  | |
| Education, *years* | 16 (2.6) | 16 (2.6) | 16 (2.5) | 16 (2.6) | 0.8^†^ | |
| BMI, *kg/m^2^* | 27.7 (4.9) | 27.8 (5.1) | 27.7 (4.6) | 27.6 (5.0) | >0.9^†^ | |
| Fecal sample BSS | 3.9 (1.2) | 3.8 (1.2) | 3.9 (1.2) | 3.9 (1.2) | 0.9^†^ | |
| Sample collection-storage interval, *days* | 1.1 (0.50) | 1.1 (0.57) | 1.1 (0.53) | 1.1 (0.36) | 0.8^†^ | |
| Unknown | 1 | 0 | 1 | 1 |  | |
| ^*^Mean (SD), n (%) | | | | | |  |
| ^†^One-way analysis of means (not assuming equal variances)  ^††^Pearson's Chi-squared test | | | | | |  |

## Heading abbreviations are defined in table S1. *BMI*, body mass index; *BSS*, Bristol Stool Scale score; *CSF*, cerebrospinal fluid; *PET*, positron emission tomography; *PiB*, ^11^C-Pittsburgh compound B. BSS ranges from 1 (hard lumps) to 7 (liquid). Diagnosis/amyloid confirmation differs significantly across biomarker subsamples (*p*=0.039) because AD Aβ+ participants were excluded from analyses involving cognitive testing.

**Table S3. Characteristics of participants with PiB PET neuroimaging.**

|  |  |  |  | ***p*-values** | | | | | |
| --- | --- | --- | --- | --- | --- | --- | --- | --- | --- |
| **Characteristic** | **CU Aβ-,  N = 55^*^** | **CU Aβ+,  N = 26^*^** | **AD Aβ+, N = 6^*^** | **Global** | **CU Aβ- vs.**  **CU Aβ+** | | **CU Aβ- vs. AD Aβ+** | **CU Aβ+ vs. AD Aβ+** | |
| Age at fecal sample, *years* | 66.49 (6.22) | 68.68 (5.96) | 71.14 (4.42) | 0.076^†^ | – | | – | – | |
| Sex |  |  |  | 0.8^††^ |  | |  |  | |
| Female | 35 (64%) | 17 (65%) | 5 (83%) |  | – | | – | – | |
| Male | 20 (36%) | 9 (35%) | 1 (17%) |  | – | | – | – | |
| *APOE* genotype |  |  |  | 0.003^††^ |  | |  |  | |
| E2-E3 | 8 (15%) | 0 (0%) | 0 (0%) |  | 0.32^††^ | | 1^††^ | 1^††^ | |
| E3-E3 | 31 (56%) | 10 (38%) | 1 (17%) |  | – | | – | – | |
| E3-E4 | 14 (25%) | 15 (58%) | 3 (50%) |  | 0.12^††^ | | 0.26^††^ | 1^††^ | |
| E4-E4 | 2 (3.6%) | 1 (3.8%) | 2 (33%) |  | 1^††^ | | 0.12^††^ | 0.26^††^ | |
| Education, *years* | 16.13 (2.75) | 15.92 (2.31) | 15.83 (1.94) | >0.9^†^ | – | | – | – | |
| BMI, *kg/m^2^* | 27.88 (5.10) | 27.89 (5.07) | 27.27 (5.36) | >0.9^†^ | – | | – | – | |
| Fecal sample BSS | 3.82 (1.12) | 3.73 (1.22) | 3.67 (1.63) | >0.9^†^ | – | | – | – | |
| PiB global cortical DVR | 1.05 (0.04) | 1.35 (0.24) | 1.67 (0.10) | <0.001^†^ | <0.001^§^ | | <0.001^§^ | <0.001^§^ | |
| Sample collection-storage interval, *days* | 1.07 (0.33) | 1.12 (0.52) | 1.67 (1.63) | 0.7^†^ | – | | – | – | |
| ^*^Mean (SD); n (%) | | | | | |  |  |  |  |
| ^†^One-way analysis of means (not assuming equal variances)  ^††^Fisher's exact test  ^§^Games-Howell test | | | | | |  |  |  |  |

Heading abbreviations are defined in tables S1 and S2. *DVR*, distribution volume ratio.

**Table S4. Characteristics of participants with cognitive testing.**

| **Characteristic** | **CU Aβ-, N = 73^*^** | **CU Aβ+, N = 30^*^** | ***p*-value** |
| --- | --- | --- | --- |
| Age at fecal sample, *years* | 66 (6.0) | 69 (6.6) | 0.015^†^ |
| Sex |  |  | >0.9^††^ |
| Female | 46 (63%) | 19 (63%) |  |
| Male | 27 (37%) | 11 (37%) |  |
| *APOE* genotype |  |  | 0.035^§^ |
| E2-E3 | 9 (13%) | 1 (3.3%) | 0.68^§^ |
| E2-E4 | 1 (1.4%) | 1 (3.3%) | - |
| E3-E3 | 42 (61%) | 13 (43%) |  |
| E3-E4 | 17 (25%) | 14 (47%) | 0.93^§^ |
| E4-E4 | 0 (0%) | 1 (3.3%) | 0.08^§^ |
| Unknown | 4 | 0 |  |
| BMI, *kg/m^2^* | 28 (5.1) | 27 (4.9) | 0.5^†^ |
| Education, *years* | 16 (2.8) | 16 (2.3) | 0.8^†^ |
| Fecal sample BSS | 3.9 (1.2) | 3.8 (1.3) | 0.6^†^ |
| Sample collection-storage interval, *days* | 1.07 (0.31) | 1.10 (0.48) | 0.7^†^ |
| Unknown | 1 | 0 |  |
| RAVLT: Trials 1-5, *words learned* | 52.65 (8.38) | 52.60 (11.63) | >0.9^†^ |
| Unknown | 1 | 0 |  |
| RAVLT: Trial 7, *words recalled* | 10.71 (2.94) | 10.53 (3.77) | 0.8^†^ |
| Unknown | 1 | 0 |  |
| TMT: Trial B Time, *seconds* | 58.60 (25.61) | 61.40 (28.14) | 0.6^†^ |
| Unknown | 1 | 0 |  |
| ^*^Mean (SD); n (%) | | | |
| ^†^One-way analysis of means (not assuming equal variances)  ^††^Pearson's Chi-squared test  ^§^Fisher's exact test | | | |

## Heading abbreviations are defined in figure S1, and tables S1 and S2. Pairwise Fisher’s exact tests performed for APOE genotype were Bonferroni-Hochberg corrected for Type I inflation error due to multiple comparisons testing.

**Table S5. Characteristics of participants with CSF biomarkers.**

|  |  |  |  | ***p*-values** | | | | | | |
| --- | --- | --- | --- | --- | --- | --- | --- | --- | --- | --- |
| **Characteristic** | **CU Aβ-, N = 54**^*^ | **CU Aβ+, N = 25**^*^ | **AD Aβ+, N = 11**^*^ | **Global** | **CU Aβ- vs. CU β+** | | **CU Aβ- vs. AD Aβ+** | | **CU Aβ+ vs. AD Aβ+** | |
| Age at fecal sample, *years* | 65 (6.2) | 68 (5.4) | 75 (4.9) | <0.001^†^ | 0.17^§^ | | 0.046^§^ | | 0.50^§^ | |
| Sex |  |  |  | >0.9 | – | | – | | – | |
| Female | 33 (61%) | 16 (64%) | 7 (64%) |  |  | |  | |  | |
| Male | 21 (39%) | 9 (36%) | 4 (36%) |  |  | |  | |  | |
| *APOE* genotype |  |  |  | 0.022^††^ |  | |  | |  | |
| E2-E3 | 8 (16%) | 1 (4.0%) | 0 (0%) |  | >0.9^††^ | | >0.9^††^ | | >0.9^††^ | |
| E2-E4 | 1 (2.0%) | 1 (4.0%) | 0 (0%) |  | 0.89^††^ | | >0.9^††^ | | >0.9^††^ | |
| E3-E3 | 27 (54%) | 9 (36%) | 2 (18%) |  | – | | – | | – | |
| E3-E4 | 13 (26%) | 12 (48%) | 7 (64%) |  | 0.40^††^ | | 0.21^††^ | | 0.89^††^ | |
| E4-E4 | 1 (2.0%) | 2 (8.0%) | 2 (18%) |  | 0.56^††^ | | 0.21^††^ | | 0.89^††^ | |
| Unknown | 4 | 0 | 0 |  |  | |  | |  | |
| BMI, *kg/m^2^* | 28 (4.5) | 28 (5.2) | 26 (3.7) | 0.4^†^ | – | | – | | – | |
| Fecal sample BSS | 3.9 (1.3) | 4.0 (0.89) | 3.8 (1.5) | >0.9^†^ | – | | – | | – | |
| Sample collection-storage interval, *days* | 1.1 (0.35) | 1.1 (0.28) | 1.5 (1.2) | 0.6^†^ | – | | – | | – | |
| Unknown | 1 | 0 | 0 |  |  | |  | |  | |
| Education, *years* | 16 (2.7) | 16 (2.0) | 14 (2.0) | 0.024^†^ | 0.61^§^ | | 0.54^§^ | | 0.85^§^ | |
| Aβ_42_/Aβ_40_ | 0.07 (0.01) | 0.04 (0.01) | 0.03 (0.01) | <0.001^†^ | <0.001^§^ | | <0.001^§^ | | 0.072^§^ | |
| pTau_181_/ Aβ_42_ | 0.02 (0.01) | 0.04 (0.02) | 0.08 (0.03) | <0.001^†^ | 0.001^§^ | | 0.058^§^ | | 0.15^§^ | |
| tTau, *pg/mL* | 192 (56) | 252 (83) | 368 (147) | <0.001^†^ | 0.076^§^ | | 0.094^§^ | | 0.18^§^ | |
| Neurogranin, *pg/mL* | 746 (254) | 952 (378) | 1175 (547) | 0.011^†^ | 0.29^§^ | | 0.20^§^ | | 0.35^§^ | |
| NFL, *pg/mL* | 91 (40) | 114 (54) | 192 (75) | <0.001^†^ | 0.25^§^ | | 0.13^§^ | | 0.30^§^ | |
| GFAP, *ng/mL* | 9.8 (3.5) | 11 (3.3) | 15 (5.4) | 0.013^†^ | 0.66^§^ | | 0.68^§^ | | 0.92^§^ | |
| YKL-40, *ng/mL* | 153 (52) | 166 (54) | 225 (94) | 0.056^†^ | – | | – | | – | |
| sTREM2, *ng/mL* | 7.9 (2.3) | 8.6 (2.6) | 9.9 (3.0) | 0.10^†^ | – | | – | | – | |
| α-Synuclein, *ng/mL* | 152 (58) | 168 (57) | 247 (105) | 0.024^†^ | 0.87^§^ | | 0.23^§^ | | 0.27^§^ | |
| S100B, *ng/mL* | 1.1 (0.20) | 1.2 (0.27) | 1.3 (0.27) | 0.052^†^ | – | | – | | – | |
| IL-6, *pg/mL* | 4.6 (3.3) | 4.6 (3.9) | 4.5 (2.8) | >0.9^†^ | – | | – | | – | |
| ^*^Mean (SD); n (%) | | | | | |  | |  | |  |
| ^†^One-way analysis of means (not assuming equal variances)  ^††^Fisher's exact test  ^§^Games-Howell test | | | | | |  | |  | |  |

Heading abbreviations are defined in tables S1 and S2. Pairwise posthoc tests were Bonferroni-Hochberg corrected for Type I inflation error due to multiple comparisons testing.

**Table S6. Diagnosis and age effects on calprotectin levels.**

| **Reference Cohort** | **Predictor** | **Estimate (log_10_ μg/g)** | ***p*-value** |
| --- | --- | --- | --- |
| $calprotectin=disease status+sex$ | | | |
| CU Aβ- | CU Aβ+ | 0.040 (-0.11, 0.20) | 0.59 |
| ***CU Aβ-*** | ***AD Aβ+*** | ***0.29 (0.080, 0.49)*** | ***0.010*** |
| ***CU Aβ+*** | ***AD Aβ+*** | ***0.24 (0.020, 0.47)*** | ***0.030*** |
| $calprotectin=disease status+sex+age$ | | | |
| CU Aβ- | CU Aβ+ | 0.010 (-0.16, 0.15) | 0.92 |
| CU Aβ- | AD Aβ+ | 0.15 (-0.069, 0.37) | 0.18 |
| CU Aβ+ | AD Aβ+ | 0.16 (-0.063, 0.38) | 0.16 |
| ***CU Aβ-*** | ***Age*** | ***0.020 (0.003, 0.031)*** | ***0.015*** |
| $calprotectin=disease status+sex+age+BMI$ | | | |
| CU Aβ- | CU Aβ+ | 0.00 (-0.16, 0.15) | 0.96 |
| CU Aβ- | AD Aβ+ | 0.17 (-0.06, 0.39) | 0.15 |
| CU Aβ+ | AD Aβ+ | 0.17 (-0.05, 0.39) | 0.13 |
| ***CU Aβ-*** | ***Age*** | ***0.020 (0.00, 0.030)*** | ***0.030*** |

## Heading abbreviations are defined in tables S1 and S2. Effects were tested using full participant cohort. β coefficients with significant *p*-values (multiple regression) are bolded and italicized.

**Table S7. Age effects on calprotectin levels within cognitively unimpaired-only cohort.**

| **Reference Cohort** | **Predictor** | **Estimate (log_10_ μg/g)** | ***p*-value** |
| --- | --- | --- | --- |
| $calprotectin=disease status$ | | | |
| CU Aβ- | CU Aβ+ | 0.040 (-0.12, 0.20) | 0.61 |
| $calprotectin=disease status+age$ | | | |
| ***CU Aβ-*** | ***Age*** | ***0.017 (0.0030, 0.031)*** | ***0.016*** |
| CU Aβ- | CU Aβ+ | -0.0090 (-0.16, 0.15) | 0.91 |
| $calprotectin=disease status+age+sex$ | | | |
| ***CU Aβ-*** | ***Age*** | ***0.017 (0.0030, 0.031)*** | ***0.021*** |
| CU Aβ- | CU Aβ+ | -0.0060 (-0.16, 0.15) | 0.94 |
| $calprotectin=disease status+age+sex+BMI$ | | | |
| ***CU Aβ-*** | ***Age*** | ***0.015 (0.0010, 0.030)*** | ***0.042*** |
| CU Aβ- | CU Aβ+ | -0.0010 (-0.16, 0.15) | 0.99 |

## Heading abbreviations are defined in tables S1 and S2. Effects were tested among cognitively unimpaired participants, comparing effects of age and CU Aβ+ status against the CU Aβ- reference cohort. β coefficients with significant *p*-values (multiple regression) are bolded and italicized.

**Table S8. Lifestyle factors compared across low- and high-calprotectin CU Aβ- participants.**

| **Characteristic** | **Low,**  **N = 69**^†^ | **High,**  **N = 10**^†^ | ***p*-value**^‡^ |
| --- | --- | --- | --- |
| Calprotectin, *log_10_(μg/g)* | 1.00 (0.27) | 1.98 (0.24) | <0.0010 |
| Age at fecal sample, *years* | 66 (6) | 67 (7) | 0.40 |
| Cardiovascular disease |  |  |  |
| Type 2 diabetes | 5 (7.2%) | 3 (30%) | 0.059 |
| Pre-diabetes | 13 (19%) | 4 (40%) | 0.20 |
| Microbiome-altering medications |  |  |  |
| Proton pump inhibitor | 5 (7.2%) | 4 (40%) | 0.013 |
| Selective serotonin reuptake inhibitor | 5 (7.2%) | 1 (10%) | 0.60 |
| Bulk-forming laxative | 6 (8.7%) | 1 (10%) | >0.90 |
| Diet |  |  | 0.70 |
| Vegetarian/vegan | 2 (1.4%) | 0 (0.00%) |  |
| Meat/fish/poultry 1-2 days weekly | 8 (12%) | 0 (0.00%) |  |
| Meat/fish/poultry 3+ days weekly | 59 (86%) | 10 (100%) |  |
| Animal caretaking |  |  |  |
| House pets | 33 (48%) | 6 (60%) | 0.50 |
| Other animals, e.g., farm animals | 8 (12%) | 1 (10%) | >0.90 |
| Early childhood microbial exposure |  |  |  |
| Cesarian section birth | 3 (4.3%) | 0 (0.00%) | >0.90 |
| Breastfeeding in infanthood | 30 (54%) | 4 (57%) | >0.90 |
| ^†^Mean (SD); n (%) | | | |
| ^‡^Wilcoxon rank sum test; Fisher's exact test | | | |

Gut microbiome-altering medications were derived from the meta-analysis by Vich Vila et al. 2020; laxatives were identified using the Anatomical Therapeutic Chemical classification system.

**Table S9. Calprotectin interaction effects with age and amyloid positivity on PiB global DVR.**

| **Estimate** | ***p*-value** |
| --- | --- |
| $DVR=calprotectin+age+\left( calprotectin\times age \right)$ | |
| -0.00060, (-0.0093, 0.0081) | 0.89 |
| $DVR=calprotectin+age+\left( calprotectin\times age \right)+sex+APOE+BMI$ | |
| -0.0056, (-0.14, 0.0024) | 0.17 |
| $DVR=calprotectin+A\beta^{+} status+\left( calprotectin\times A\beta^{+} status \right)$ | |
| -0.026, (-0.19, 0.24) | 0.81 |
| $DVR=calprotectin+A\beta^{+} status+\left( calprotectin\times A\beta^{+} status \right)+age+sex+APOE+BMI$ | |
| -0.051, (-0.15, 0.26) | 0.62 |

Calprotectin-by-age effects were estimated using the full study cohort (N=125), and calprotectin-by-amyloid positivity effects were estimated in the CU cohort (N=112). Effects were estimated using multiple regression and were nonsignificant before adjusting for covariates.

**Table S10. Calprotectin relationships with CSF biomarkers.**

| **Biomarker** | **Estimate** | ***p-*value** | ***q-*value** |
| --- | --- | --- | --- |
| ***Aβ_42_/Aβ_40_*** | -0.0010, (-0.0090, 0.0070) | 0.80 | 0.82 |
| ***pTau_181_/Aβ_42_*** | -0.0040, (-0.016, 0.0090) | 0.57 | 0.61 |
| tTau, *pg/mL* | -51.057, (-98.96, -3.15) | 0.037 | 0.08 |
| ***NFL, pg/mL*** | -22.32, (-44.86, 0.21) | 0.052 | 0.11 |
| Neurogranin, *pg/mL* | -192.26, (-394.38, 9.86) | 0.062 | 0.12 |
| α-Synuclein, *ng/mL* | -43.58, (-82.35, -4.81) | 0.028 | 0.070 |
| sTREM2, *ng/mL* | -1.051, (-2.49, 0.39) | 0.15 | 0.23 |
| YKL-40, *ng/mL* | -52.25, (-80.80, -23.71) | 0.00033 | 0.0013 |
| GFAP, *ng/mL* | -1.42, (-3.73, 0.90) | 0.23 | 0.30 |
| S100B, *ng/mL* | -0.11, (-0.27, 0.042) | 0.16 | 0.23 |
| IL-6, *pg/mL* | 1.99, (-0.91, 4.91) | 0.18 | 0.27 |

# β coefficients were estimated using the multiple regression equation $biomarker=calprotectin+age+disease status$ (*q*, false discovery rate corrected). Bolded biomarkers were significantly associated with calprotectin before controlling for age and disease status.
